# Glucose Levels as a Mediator of the Detrimental Effect of Abdominal Obesity on Relative Handgrip Strength in Older Adults

**DOI:** 10.3390/jcm9082323

**Published:** 2020-07-22

**Authors:** Miguel Ángel Pérez-Sousa, Jesús del Pozo-Cruz, Carlos A. Cano-Gutiérrez, Atilio J. Ferrebuz, Carolina Sandoval-Cuellar, Mikel Izquierdo, Paula A. Hernández-Quiñonez, Robinson Ramírez-Vélez

**Affiliations:** 1 Faculty of Sport Sciences, University of Huelva, Avenida de las Fuerzas Armadas s/n 21007, 21004 Huelva, Spain; perezsousa@gmail.com; 2 Department of Physical Education and Sport, University of Seville, 41092 Seville, Spain; jpozo2@us.es; 3 Hospital Universitario San Ignacio-Aging Institute, Pontificia Universidad Javeriana, Bogotá 110111, Colombia; ccano@javeriana.edu.co; 4 Facultad de Ciencias de la Salud, Universidad de Boyacá, Tunja 150003, Colombia; ajferrebuz@uniboyaca.edu.co (A.J.F.); carolinasandoval@uniboyaca.edu.co; (C.S.-C.); 5 Navarrabiomed, Complejo Hospitalario de Navarra (CHN)-Universidad Pública de Navarra (UPNA), IdiSNA, 31008 Pamplona, Spain; mikel.izquierdo@gmail.com; 6 CIBER of Frailty and Healthy Aging (CIBERFES), Instituto de Salud Carlos III, 28029 Madrid, Spain.; 7 GICAEDS Group, Faculty of Physical Culture, Sport and Recreation, Universidad Santo Tomás, Bogotá 110311, Colombia; paulahernandezq@usantotomas.edu.co

**Keywords:** fat mass, obesity, muscle strength, physical function, diabetes

## Abstract

Excess central adiposity accelerates the decline of muscle strength in older people. Additionally, hyperglycemia, independent of associated comorbidities, is related to the loss of muscle mass and strength, and contributes to functional impairment in older adults. We studied the mediation effect of glucose levels, in the relationship between abdominal obesity and relative handgrip strength (HGS). A total of 1571 participants (60.0% women, mean age 69.1 ± 7.0 years) from 86 municipalities were selected following a multistage area probability sampling design. Measurements included demographic and anthropometric/adiposity markers (weight, height, body mass index, and waist circumference). HGS was measured using a digital dynamometer for three sets and the mean value was recorded. The values were normalized to body weight (relative HGS). Fasting glucose was analyzed by enzymatic colorimetric methods. Mediation analyses were performed to identify associations between the independent variable (abdominal obesity) and outcomes (relative HGS), as well as to determine whether fasting glucose levels mediated the relationship between excess adiposity and relative HGS. A total of 1239 (78.8%) had abdominal obesity. Abdominal obesity had a negative effect on fasting glucose (β = 9.04, 95%CI = 5.87 to 12.21); while fasting glucose to relative HGS was inversely related (β = −0.003, 95%CI = −0.005 to −0.001), *p* < 0.001. The direct effect of abdominal obesity on relative HGS was statistically significant (β = −0.069, 95%CI = −0.082 to −0.057), *p* < 0.001. Lastly, fasting glucose levels mediates the detrimental effect of abdominal obesity on relative HGS (indirect effect β = −0.002, 95%CI = −0.004 to −0.001), *p* < 0.001. Our results suggest that the glucose level could worsen the association between abdominal obesity status and lower HGS. Thus, it is plausible to consider fasting glucose levels when assessing older adults with excess adiposity and/or suspected loss of muscle mass.

## 1. Introduction

Aging is related to a progressive unfavorable change in body composition, particularly abdominal fat accumulation and loss of lean mass [1,2]. Abdominal obesity, measured by waist circumference (WC) [3], is associated with systemic inflammation, hyperlipidemia, cardiovascular diseases, impaired fasting glucose, prediabetes, insulin resistance, hyperinsulinemia, and type 2 diabetes (T2DM) [4,5,6,7,8,9,10,11,12,13,14]. Specifically, hyperglycemia, independent of associated comorbidities, is related to the loss of muscle mass and strength, and contributes to functional impairment in older adults [9,10,11,12,13]. Lower muscle mass is positively associated with central adiposity and an increased risk of developing T2DM [8]. Hyperglycemia and the presence of insulin resistance may increase autophagy, muscle protein degradation, and mitochondrial dysfunction, which may negatively impact skeletal muscle function [6]. Therefore, the coexistence of aging and abdominal obesity creates the harmful environment for the deterioration of muscle mass. On the other hand, it has been suggested that excessive and naturally occurring deposition of adipose tissue in the abdomen may increase the risk of hyperinsulinemia, metabolic syndrome, and type 2 T2DM [6,7,8].

Handgrip strength (HGS) is a simple and reliable tool for measuring body function and has been suggested as a biomarker for older adults [15]. In the past decade, the majority of studies have used the HGS normalized to body weight or body mass index, since has been recommended in the research of muscle health [16,17]. In this sense, some evidence that relative handgrip strength is associated with persistent hyperglycemia [18,19]. Joule et al. [20] found that upper muscle strength (measured by bench press) was weaker in patients with T2DM than in healthy controls, and similarly, Mee-Ri et al. [21] recently found an inverse relationship between T2DM and HGS. In older adults (> 65 years), hyperinsulinemia increases the risk of falls, dementia, depression, and vision and hearing loss [22], and is associated with a substantial burden of cardiovascular disease [23], and brain abnormalities [24], with significant long-term morbidity and mortality [13]. It seems to be that higher level on glucose also affects skeletal musculature (diabetic myopathy), involving contractile weakness, mitochondrial dysfunction, fiber-type changes, slow-to-fast muscle transitions, and decreased oxidative activity [25,26]. In addition to this, it has a negative impact on muscular strength and quality in older adults. Park et al. [27,28] found a decline in muscle strength in the lower body in older adults with T2DM. However, these and other studies have not examined muscular strength in lower or upper extremities in individuals with disorders of glucose tolerance [29,30].

Overall, these findings indicate the coexistence of two vectors negatively affecting muscle strength–excess central adiposity and higher level of glucose. However, the exact biological mechanisms are poorly understood. Nevertheless, changes in body composition, particularly declines in lean body mass and the concurrent fat accumulation, coupled with impairment glucose metabolism have been proposed as potential mediators contributing to the declines in muscle strength and quality. Because an increase in body weight (adiposity) typically precedes the development of T2DM, research examining the relationship between HGS and body weight is desirable to know more about how relative HGS is associated with central adiposity, and to test whether fasting glucose has an effect on the relationship between central adiposity and relative HGS. To date, the potential role of fasting glucose in attenuating or modifying the relationship between central adiposity and hepatic relative HGS remains unknown and, to the best of our knowledge, has not been examined in older subjects.

The interplay between sarcopenia and excess adiposity in an ageing population has now emerged as an important public health concern in older populations. Considering the increasing number of obese older adults occurring in parallel with a greater prevalence of declines in muscle strength and quality, this study aimed to investigate the possible mediation effect of fasting glucose on the relationship between central adiposity and relative HGS, in community-dwelling older adults.

## 2. Materials and Methods

### 2.1. Study Design and Sample Population

We used the database from the “Health and Well-being and Aging Survey in Colombia, 2015” (SABE, from initials in Spanish: SAlud, Bienestar & Envejecimiento, 2015), a cross-sectional study supported by the Epidemiological Office of the Ministry of Health and Social Protection of Colombia (https://www.minsalud.gov.co/) of a nationally representative sample of Colombian older adults. The sampling design in the SABE study consists of a multistage probability sampling design for participant selection according to the existing municipal cartography as municipalities, urban, rural segments, homes or sidewalks, homes, and people. Therefore, it constitutes 99% of the population residing in private homes in the urban and rural strata of the sample. The study protocol was approved by the Human Subjects Committee for this secondary analysis at the Pontificia Universidad Javeriana (ID protocol 20/2017–2017/180, FM-CIE-0459-17) following the tenets of the Declaration of Helsinki of the World Medical Association and Resolution 8430 of 1993 of the then Ministry of Health of Colombia on technical, scientific and administrative standards for human research. All participants provided written informed consent. Details of the survey have been published [31].

**Figure 1:**
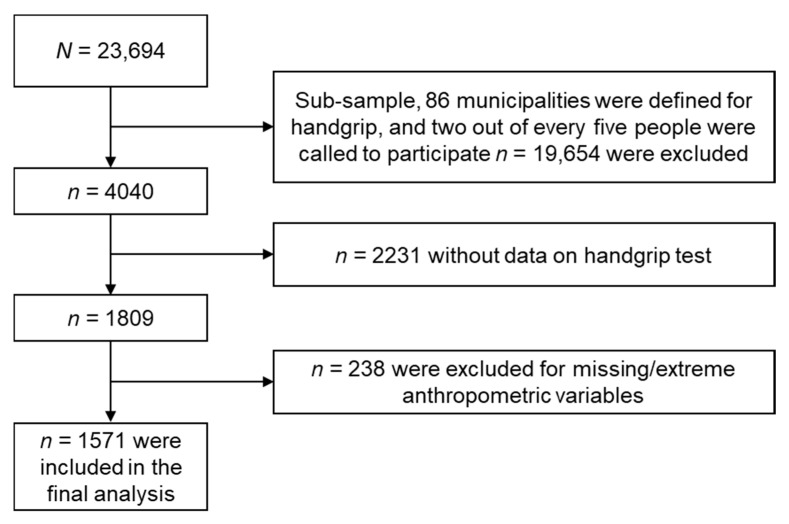
Flow chart showing the selection of the study sample from the Colombian Health and Wellbeing and Aging Survey (SABE) 2015. All analyses presented in this paper are based on 1571 surveyed participants, each with complete anthropometric, blood-based, and covariable data.

The SABE assessed 23,694 elderly people from 246 municipalities across all departments of the country. For this subsample, we selected 86 municipalities, including the four large cities. The sample size calculation was carried out, selecting two out of five individuals of the general sample, obtaining a sample of 1571 participants (60.0% women) aged 60 years and over. We included individuals who completed the handgrip strength test and who had available anthropometric/biological data to establish relative handgrip strength (Figure 1).

### 2.2. Measurements

Trained staff investigators carried out the physical examination, and medical laboratory technicians performed the blood samples and laboratory tests. With the aim to minimize the error, all analysis were performed by the Universities of Caldas and Valle, Colombia. Height and body weight were measured with a portable stadiometer (SECA 213, Hamburg, Germany) and an electronic scale (Kendall graduated platform scale). BMI was calculated in kg/m^2^ from the measured body weight and height. WC was measured over the midpoint between the lower border of the ribs and iliac crest in the midaxillary plane, at the end of normal expiration. We used WC as proxy measures of central adiposity since they are useful tools in clinical practice, and are reliable predictors of T2DM and visceral adiposity [32]. The HGS of both hands was measured with a digital hand dynamometer (Takei; Scientific Instruments Co., Ltd., Tokyo, Japan). Each participant completed the 3-trial for each hand, and the final estimate of HGS was the average of all measurements. The values were normalized to body weight (relative HGS). After an overnight fast, blood samples were obtained in the morning. Plasma glucose was analyzed by enzymatic colorimetric methods (Dinamica Laboratories, Bogotá, Colombia).

The following detailed demographics were recorded: age, sex, ethnicity, socioeconomic status (for lifestyle characteristics), alcohol intake (participants were categorized as those who do not drink and those who drink less than 1 day per week, 2 to 6 days a week, or every day), and cigarette smoking (participants were categorized as those who do not smoke and those who have never-smoked, those who currently smoke, or those who previously smoked) were recorded. A “proxy physical activity” report was conducted using questions: (i) “Have you regularly exercised, such as jogging or dancing, or performed rigorous physical activity at least three times a week for the past year?”; (ii) “Do you walk, at least three times a week, between 9 and 20 blocks (0.6 to 1.2 km) without resting?”; (iii) “Do you walk, at least three times a week eight blocks (0.5 km) without resting?”. Participants were considered physically active if they responded affirmatively to two of the three questions. Demographics such as sex, age, socioeconomic status (low, middle, and high), ethnicity (people belonging to various indigenous groups, i.e., Ika, Kankuamo, Emberá, Misak, Nasa, Wayuu, Awuá, Mokane, etc., black “mulato” or Afro-Colombian, white and others, i.e., mestizo, gypsy, etc.), and urbanicity (urban, rural) were obtained by structured interview.

### 2.3. Statistical Analysis

**Figure 2:**
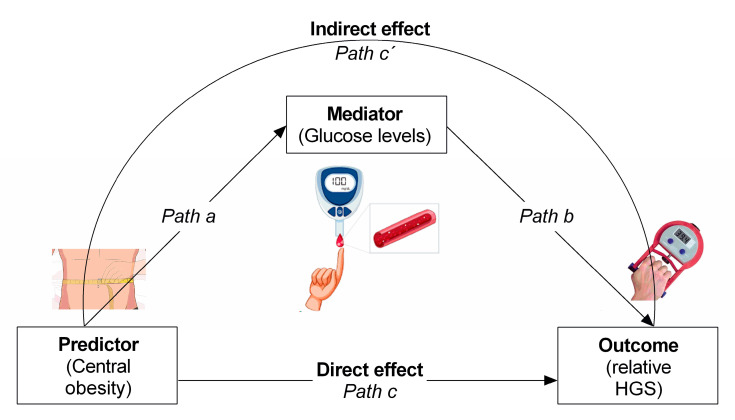
Mediation analysis tests a hypothetical causal chain where one variable X (abdominal obesity status) affects a second variable M (fasting glucose levels) and, in turn, that variable affects a third variable Y (HGS, relative handgrip strength).

Descriptive analyses of the study population characteristics were performed through mean ± standard deviation (SD) for the continuous variables and frequency distribution for categorical variables. The normality of the data was examined by the Kolmogorov–Smirnov test. Significant differences between men and women were analyzed using Student’s t-test or the chi-square (χ^2^) post-hoc test. To elucidate the differences after controlling confounder variables like sex, age, lifestyle, and sociodemographic characteristics, we performed an analysis of covariance (ANCOVA). Differences were interpreted through Cohen’s effect size indices as small (*d* = 0.2), medium (*d* = 0.5), or large (*d* = 0.8) based on benchmarks suggested by Cohen [33]. Mediation analysis was conducted to determine the indirect effect of fasting glucose levels on the relationship between abdominal obesity by WC and relative HGS (see Figure 2).

In this order, we obtained the direct effect from variable X (categorized as 0 = healthy vs. 1 = abdominal obesity) to Y (relative HGS). Fasting glucose levels were used to know whether it played a mediator role. That is, to know if the detrimental effect of being abdominal obese on poorer muscle health is mediated by fasting glucose. Note that mediator in this case would be understood as the harmful ingredient for such a damaging relationship between abdominal obesity status and muscle health to take place. The analyses were conducted using the PROCESS macro for SPSS, version 3.4.1, developed by Hayes [34]. This method provided an estimation of both the direct (Path c) and indirect (Path c’) pathways, resulting in the calculation of 95% confidence intervals (CI) for both direct and indirect effects (see Figure 2 for model depiction). The regression coefficients are displayed in unstandardized form, as the bootstrapped CI’s correspond to the unstandardized effects rather than the standardized effects (β). Mediation results are considered significant if the CI’s do not contain 0. A *p*-value < 0.05 was interpreted as statistically significant.

## 3. Results

Of the 1571 subjects included in the sample, 1239 (78.8%) had abdominal obesity and 331 were healthy (22.2%). Healthy individuals presented a mean age of 70.5 (8.1) years and abdominal obese individuals 69.6 (7.3) years (see Table 1). Statistical differences (*p* < 0.05) between groups were found for all anthropometric characteristics and glucose level with higher values for older adults with central obesity. Healthy individuals presented better performance in muscular strength than obese individuals. Also, statistical differences were found between healthy and obese individuals on ethnicity, socioeconomic status, lifestyle outcomes and urbanicity, *p* < 0.05.

Thus, to clarify the differences between central obesity status (“healthy” vs. “abdominal obesity”) adjusted by the confounder variables we performed analysis of covariance, Table 2. The ANCOVA shows that differences in glucose levels and relative HGS parameters between older adults with and without abdominal obesity were independent after adjusting for sex and age (*p* < 0.001, Model 1), sex, age, and lifestyle (*p* < 0.001, Model 2), and ANCOVA Model 2 was additionally adjusted with socioeconomic status, ethnicity, and urbanicity (*p* < 0.001, Model 3).

**Figure 3:**
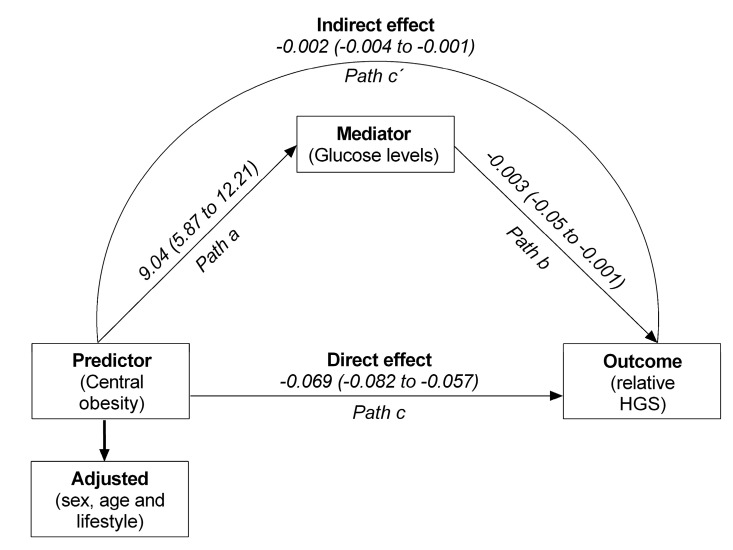
The direct effect of abdominal obesity status (healthy versus unhealthy) on relative HGS gives through fasting glucose level. In the model, abdominal obesity has an inverse relationship with relative HGS. This relationship is mediated by fasting glucose level as the active ingredient (in this case, harmful ingredient). The indirect effect is statistically significant at the 95% confidence interval (CI) when the CI does not include zero.

Figure 3 shows the results of mediation analysis to test whether the fasting glucose could be a mediator of the relationship between abdominal obesity and relative HGS. Path a indicated that central obesity status had a statistical significant negative effect on fasting glucose (*β* = 9.04, 95% CI = 5.87 to 12.21), *p* < 0.001; the *path b* from fasting glucose to relative handgrip strength was inversely related (*β* = −0.003, 95%CI −0.005 to −0.001), *p* < 0.001. The direct effect of abdominal obesity on relative HGS was statistically significant (*β* = −0.069, 95% CI = −0.082 to −0.057), *p* < 0.001. Besides, there was a significant indirect effect since the CI did not include zero. Finally, fasting glucose mediates the detrimental effect of abdominal obesity on relative HGS (*β* = −0.002, 95% CI = −0.004 to −0.001), *p* < 0.001.

## 4. Discussion

In a cross-sectional study of community-dwelling older adults, we found that central obesity was inversely associated with relative HGS, as a measure of muscular strength, in older Colombian adults. This association was mediated by fasting glucose levels. In the same line, we also showed that the abdominal obesity was associated with higher fasting glucose levels. As far as we know, this is the first study to have examined the mediation role of fasting glucose level for the relationship between abdominal obesity and relative HGS. Our results suggest that the glucose level could worsen the association between abdominal obesity status and lower relative HGS. Also, our results provide novel insight into the mechanisms underlying this relationship.

In this study, WC were used as proxy measures for abdominal obesity which have been widely used in older adults for identification of central obesity in older adults [35]. According to the IDF guidelines cutoff point of WC for abdominal obesity, presents a reliable measure of visceral fat [3] in Latin-American people and is strongly associated with metabolic syndrome. Our findings clearly showed that abdominal obesity is associated with a low level of relative HGS. Our results are consistent with previous literature showing that obesity, particularly central adiposity, is inversely associated with strength and/or muscle quality in older adults [8]. Also, we found the relationship between WC and impaired fasting glucose, which is in agreement with previous research showing that central adiposity measured by WC is strongly related to more incidents of T2DM [8,35,36].

In a review study, Freemantle et al. [36] found that WC was strongly associated with T2DM, and Wang et al. [37] also found that WC was a better predictor of T2DM than BMI, even in non-obese individuals. Likewise, Son et al. [38] found a strong association between waist-to-height ratio (another proxy marker for central obesity dysfunction) and hyperglycemia. Overall, these findings support a clinically relevant issue which, through a simple measure of central adiposity, could help to screen for chronic metabolic disorder.

In our study, also showed that higher level of fasting glucose was inversely associated with relative HGS. According to the Guidelines on Integrated Care for Older People, handgrip strength is considered a reliable tool for measuring muscular fitness in older adults [39]. Low relative HGS is an indicator of poor physical performance [40], and it is clear that low levels of physical fitness are related to a lower level of muscle mass [41]. Our results are consistent with previous research which found an inverse association between muscle strength and impaired fasting glucose [21,42,43].

The key finding of this study was that fasting glucose plays a mediator role in the relationship between abdominal obesity and relative HGS. To the best of our knowledge, this is the first study investigating this hypothesis in older adults. Other studies show that fatness is a mediator of muscular fitness and metabolic syndrome [44] in adolescents and, similarly, Brand et al. [45] and Bailey et al. [46] found that body fat mediated the relationship between cardiorespiratory fitness and cardiovascular risk factors. Also, it has been shown that patients with T2DM and with visceral fat accumulation have low muscle quality [47]. Although we did not perform measures of muscle quality per se, previous evidence is suggestive that total and regional adiposity is associated with inter- and intramuscular adipose tissue infiltration, which is considered to be an important anatomical correlation of poor muscle quality [48]. Additionally, the aging effect since age is linked to increased body fat accumulation, insulin resistance, and muscle strength decline [48]. Several epidemiological studies have previously reported that skeletal muscle fat infiltration with age is associated with a decrease in muscle density, loss of muscle quality, poor lower body extremity performance, and falls risk [16,17,18,19]. In the same line, higher level of fasting glucose mediating detrimental effect of abdominal obesity on muscle strength, might be the result of a greater content of glucose causing muscle atrophy [49,50]. Skeletal muscle seems to be a protector against diabetes [51]. Mechanistically, this might involve better insulin clearance by muscle myocytes. In this line, it has been shown that an insulin molecule activated by an insulin receptor in the muscle offers 2.1- to 3.1-times higher glucose uptake (removal) than the same insulin molecule activated by an insulin receptor in an adipocyte [52]. Another protector role of muscle mass is better glycolysis by increased glucose transport via GLUT-4 expression from intracellular pools to the surface cell membrane [53,54]. Therefore, individuals who are fit display higher insulin sensitivity than unfit, obese, or sarcopenic individuals [55].

As indicated above, an increase in intramuscular fat could lead to insulin resistance due to the presence of adipocytes, which worsens glucose clearance [52]. Additionally, this effect leads to a worsening of the intramuscular mitochondrial function since the concomitant atrophy reduces the oxidative and phosphorylation activity of muscle mitochondria [56]. Furthermore, the underlying functions of cytokines and myokines might come into play in this environment. It has been shown that the production of proinflammatory cytokines may be one of the crucial mechanisms for T2DM development as, without good muscle health, the anti-inflammatory myokines cannot prevent systemic inflammation and development of T2DM [57,58].

The attributable risk for chronic metabolic disorder associated with low HGS has been previously reported from populations with varying ethnic backgrounds in different settings within one region or country [20,59,60]. In this line, our findings are consistent with prior reports in the literature as a number of previous studies have emphasized that HGS is inversely associated with plasma glucose after adjusting for age, sex, and BMI [61]. Peterson et al. [43] reported that every 0.05 kg decrease in the relative HGS was independently associated with a 1.49 (95% CI: 1.42–1.56) and 1.17 (95% CI: 1.11–1.23) odds for T2DM in American and Chinese adults, respectively, while among older Mexican Americans, muscle weakness was associated with T2DM (hazard ratio: 1.05; 95% CI: 1.02–1.09). [19] Notably, we found that glucose fasting plays a mediator role in the negative effect of abdominal obesity on relative HGS in Colombian older adults. However, since this research used a cross-sectional design, casual relationships cannot be inferred. The precise mechanisms for the observed associations must be examined in future studies.

Therefore, primary care strategies should be developed to prevent the loss of muscle mass and muscular strength [62]. Also, maintaining low body fat could help to avoid the deterioration of muscular health associated with insulin resistance or pre-diabetes. These findings can help guide physical exercise programs for coaches, sports technicians or health agents, and nutritionists, prioritizing physical exercise and diet to reduce the accumulation of fat.

Our study has several limitations, including its cross-sectional design, which prevents us from making causal inferences [50]. However, the strengths of this study are the mediation analysis that, to our knowledge, is the first to study the role of glucose levels in the relationship between central adiposity and muscular strength in Latin-American older adults. Also, our results are comparable with other health surveys since both muscle strength and abdominal adiposity were measured using simple and reliable tools for clinical practice. Therefore, the results of this study can provide a foundation for developing hypotheses for longitudinal studies.

## 5. Conclusions

In summary, fasting glucose level mediates the association between abdominal obesity status and relative HGS in Colombian older adults. Our findings illustrate the importance of glucose control and healthy habits for the prevention of insulin resistance in older with abdominal obesity and the relevance of optimum muscular strength. Longitudinal studies are required in the future to further clarify the influence of glucose levels on this relationship in community-dwelling older adults.

## Figures and Tables

**Table 1:** Characteristics of the study participants (*n* = 1571).

Characteristics	Full Sample (*n* = 1571)	Healthy (*n* = 331)	Abdominal Obesity (*n* = 1239)	*p*-Value
Age, years	69.6 (7.3)	70.5 (8.1)	69.6 (7.3)	0.052
Sex, *n* (%)				
Females	943 (60.0)	108 (32.6)	835 (67.3)	<0.001
Clinical outcomes, mean (SD)				
Body mass, kg	68.3 (11.5)	55.2 (8.4)	68.3 (11.5)	<0.001
Height, m	1.55 (0.08)	1.59 (0.08)	1.55 (0.07)	<0.001
BMI, Kg/m^2^	28.9 (4.3)	22.5 (2.7)	28.9 (4.3)	<0.001
Waist circumference, cm	96.1 (9.1)	79.3 (6.6)	96.1 (9.1)	<0.001
Glucose fasting, mg/dL	100.1 (26.3)	90.4 (18.0)	100.1 (26.3)	<0.001
Muscular strength, mean (SD)				
HGS (kg)	21.1 (8.4)	22.9 (8.6)	20.6 (8.2)	<0.001
Relative HGS (kg/kg body mass)	0.32 (0.12)	0.41 (0.13)	0.30 (0.1)	<0.001
Race/ethnic group, *n* (%)				
Indigenous	79 (5.0)	21 (6.3)	58 (4.7)	<0.001
Black “mulato” or Afro-Colombian	125 (8.0)	32 (9.7)	93 (7.5)	<0.001
White	414 (26.4)	70 (21.1)	344 (27.7)	<0.001
Others *	753 (47.9)	153 (46.2)	600 (48.4)	<0.001
Missing date	200 (12.7)	55 (16.6)	145 (11.7)	―
Socioeconomic status, *n* (%)				
Level I-II (low)	1138 (72.4)	247 (74.6)	891 (71.9)	<0.001
Level III-IV (middle)	424 (27.0)	83 (25.1)	341 (27.5)	<0.001
Level V-VI (high)	9 (0.6)	1 (0.3)	8 (0.6)	0.020
Lifestyle outcomes, *n* (%)				
Smoking	152 (9.7)	56 (16.9)	96 (7.7)	0.001
Alcohol intake	203 (12.9)	59 (17.9)	144 (11.6)	<0.001
Physical activity “proxy”	1278 (81.3)	261 (78.9)	1017 (82.2)	<0.001
Urbanicity, *n* (%)				
Urban	1311 (83.5)	247 (74.6)	1064 (85.8)	<0.001
Rural	260 (16.5)	84 (25.4)	176 (14.2)	<0.001

Data are presented as mean ± SD or number (percentage) of participants. Significant differences between groups were analyzed by Student’s *t*-test or χ^2^ test. BMI: body mass index; * Others (mestizo, gypsy, etc.).

**Table 2:** Comparison of the marginal mean values of fasting glucose levels and relative HSG according to central obesity status.

Variables	Model 1			Model 2			Model 3		
	Healthy	Abdominal Obesity	*d*	Healthy	Abdominal Obesity	*d*	Healthy	Abdominal Obesity	*d*
Glucose levels (mg/dl)	90.5 (87.8; 93.3)	100.0 (98.6; 101.4)	0.38 *	90.9 (88.1; 93.7)	99.9 (98.5; 101.3)	0.36 *	90.9 (87.8; 94.0)	100.1 (98.5; 101.5)	0.36 *
Relative HSG (kg/kg)	0.38 (0.37; 0.39)	0.30 (0.29; 0.31)	0.65 *	0.38 (0.37; 0.39)	0.31 (0.30; 0.32)	0.63 *	0.38 (0.37; 0.39)	0.31 (0.30; 0.32)	0.62 *

Data are presented as mean and (95% CI), *d* = Cohen’s effect size, * *p* < 0.001.
